# Among-Population Variation in Microbial Community Structure in the Floral Nectar of the Bee-Pollinated Forest Herb *Pulmonaria officinalis* L

**DOI:** 10.1371/journal.pone.0056917

**Published:** 2013-03-11

**Authors:** Hans Jacquemyn, Marijke Lenaerts, Rein Brys, Kris Willems, Olivier Honnay, Bart Lievens

**Affiliations:** 1 Division of Plant Ecology and Systematics, Biology Department, KU Leuven, Heverlee, Belgium; 2 Laboratory for Process Microbial Ecology and Bioinspirational Management, Thomas Moore University College, Campus De Nayer, Department of Microbial and Molecular Systems (M2S), KU Leuven Association, Sint-Katelijne-Waver, Belgium; 3 Scientia Terrae Research Institute, Sint-Katelijne-Waver, Belgium; Dowling College, United States of America

## Abstract

**Background:**

Microbial communities in floral nectar have been shown to be characterized by low levels of species diversity, yet little is known about among-plant population variation in microbial community composition.

**Methodology/Principal Findings:**

We investigated the microbial community structure (yeasts and bacteria) in floral nectar of ten fragmented populations of the bee-pollinated forest herb *Pulmonaria officinalis*. We also explored possible relationships between plant population size and microbial diversity in nectar, and related microbial community composition to the distance separating plant populations. Culturable bacteria and yeasts occurring in the floral nectar of a total of 100 plant individuals were isolated and identified by partially sequencing the 16S rRNA gene and D1/D2 domains of the 26S rRNA gene, respectively. A total of 9 and 11 yeast and 28 and 39 bacterial OTUs was found, taking into account a 3% (OTU_0.03_) and 1% sequence dissimilarity cut-off (OTU_0.01_). OTU richness at the plant population level (i.e. the number of OTUs per population) was low for yeasts (mean: 1.7, range: 0–4 OTUs_0.01/0.03_ per population), whereas on average 6.9 (range: 2–13) OTUs_0.03_ and 7.9 (range 2–16) OTUs_0.01_ per population were found for bacteria. Both for yeasts and bacteria, OTU richness was not significantly related to plant population size. Similarity in community composition among populations was low (average Jaccard index: 0.14), and did not decline with increasing distance between populations.

**Conclusions/Significance:**

We found low similarity in microbial community structure among populations, suggesting that the assembly of nectar microbiota is to a large extent context-dependent. Although the precise factors that affect variation in microbial community structure in floral nectar require further study, our results indicate that both local and regional processes may contribute to among-population variation in microbial community structure in nectar.

## Introduction

Floral nectar is a sweet, aqueous secretion containing sugars and amino acids that is offered by flowering plants to attract pollinators, mostly insects [1], [2]. Traditionally it has been assumed that nectar properties represent intrinsic plant features that are stable in time. However, recent studies have indicated that nectar is often contaminated with microorganisms, most often yeasts and bacteria, which may change the chemistry and attractiveness of nectar, potentially affecting pollination success and plant fitness [3], [4]. Although it has already been known since the early 1920’s that yeasts are common inhabitants of floral nectars [5], [6], only recently the microbial community structure in nectar and its ecological impact have been explored in more detail [7]–[12]. These studies have highlighted that the floral nectar of animal-pollinated plants often harbors highly specialized yeast communities. In most cases, species richness tends to be low and often only a few species are found within the nectar of a single nectary [8], [12], [13], suggesting that important filtering mechanisms (e.g. priority effects and nectar chemistry) determine community composition of nectar-inhabiting microorganisms in floral nectar [11], [14], [15].

Bacteria, on the other hand, have been less frequently studied, and there are only a few studies that have characterized bacterial communities in floral nectar [16], [17]. A recent study, investigating phylogenetic diversity of culturable bacteria in 27 South African plant species, revealed that bacteria are common in floral nectar, but that their phylogenetic diversity is rather restricted, with most isolates belonging to three major bacterial phyla, including *Actinobacteria*, *Firmicutes*, and *Proteobacteria* (*Alphaproteobacteria*, *Betaproteobacteria* and *Gammaproteobacteria*) [18]. Similar to yeast communities, species richness was also found to be low (18 operational taxonomic units (OTUs) at a 16S rRNA gene sequence dissimilarity cut-off of 3%) (but see [19]).

Given the mounting number of studies that have investigated microbial community composition in floral nectar [7], [9], [10], [12], [18]–[22], surprisingly little is known about among-plant population variation in community composition of these nectar-inhabiting microorganisms. Although precipitation and microorganisms in the air can be considered as constant sources of microorganisms in flowers, yeasts and bacteria are most likely transported to flowers by pollinating insects or small birds [9], [14], [15]. It is therefore reasonable to assume that limitations in their dispersal capacity may lead to significant spatial turnover of microbial community composition in floral nectar, especially when plant populations occur in highly fragmented habitats, surrounded by an inhospitable urban or agricultural landscape matrix. Recent research investigating community organization of nectar-inhabiting microorganisms in the hummingbird-pollinated shrub *Mimulus aurantiacus* has indeed shown significant turnover of microbial community composition, even at a very small scale [14]. However, there is currently no information available regarding differences in microbial community structure among plant populations that occur in discrete habitat fragments within a hostile matrix.

In this paper, we investigated the community structure of nectar-inhabiting microorganisms in ten fragmented populations of the bee-pollinated understory forest herb *Pulmonaria officinalis* (Common lungwort) in northern Belgium. Previous genetic marker based research on this plant species in the same area has shown strong genetic differentiation and significant isolation-by-distance [23]. Because gene flow in this species occurs mainly through pollen [24], these results indicate that pollen dispersal is mainly restricted to neighboring populations. Assuming that pollinators are the main dispersal agents of nectar-inhabiting microorganisms [7] and given that local populations occur in forest fragments with pronounced differences in local environmental conditions, it can be expected that community composition of nectar-inhabiting microorganisms is more dependent on population characteristics, such as nectar quality, population size or local plant community composition, than on geographic isolation. As a result, similarity in community composition between populations is expected to be low. To test these general predictions, the presence of culturable yeasts and bacteria was assessed for each population and microbial species richness at the plant population level was related to the size of the plant population. Finally, similarity in microbial community composition between populations was assessed and related to the distance between plant populations.

## Materials and Methods

### Ethics Statement

All necessary permits were obtained for the described field studies.

### Study Species

**Figure 1:**
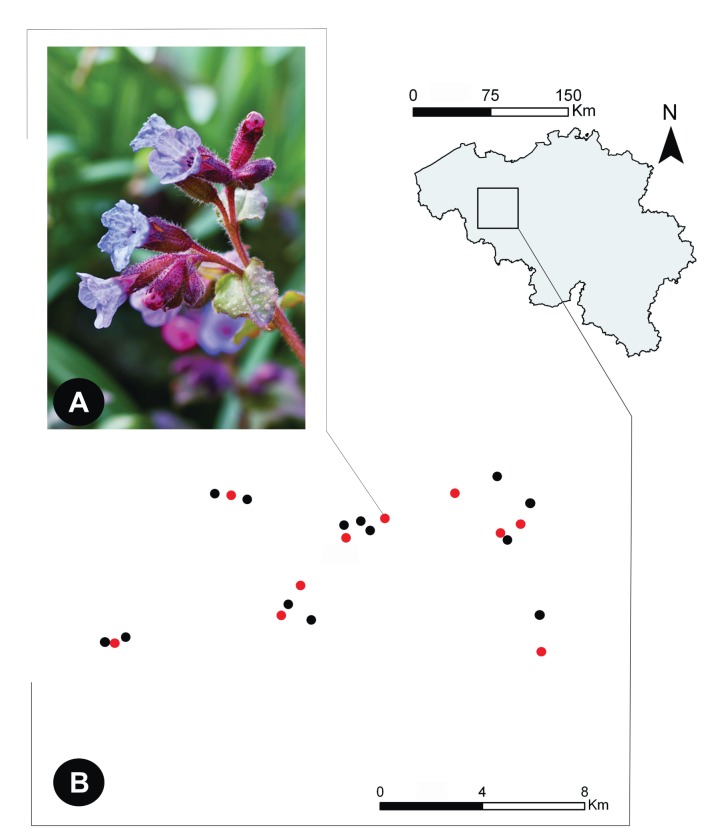
Geographic distribution of ten populations of the bee-pollinated forest herb *Pulmonaria officinalis* used in this study (indicated in red) among several other *P. pulmonaria* populations in the same study area (northern Belgium).

*Pulmonaria officinalis* L. is a perennial forest herb that grows in species-rich mixed and open forests, characterized by relatively humid, wet and loamy soils. Its distribution range is located in Mid-East Europe, but fragmented populations reach till Britain and Denmark. The species is wintergreen and flowers early in the growth season, from March until the end of April. Flowers exhibit reciprocal herkogamy and several ancillary polymorphisms [25]. During anthesis, the colour of the corolla gradually changes from red through purple to violet and finally blue, offering a visual sign to pollinators which flowers are most rich in nectar [26]. Nectar is secreted at the bottom of the corolla tube, where it accumulates. Within the study area (northern Belgium, Flanders, Fig. 1), flowers are visited by generalist insect species, including *Bombus terrestris*, *B. pascuorum*, *B. pratorum* and *Bombylius major*, but only the long-tongued *Anthophora plumipes* was shown to serve as an efficient pollinator [25].

### Study Region and Nectar Sampling

This study was conducted in the region around Brakel, where several *P. officinalis* populations are known to occur in small, highly fragmented forest patches [23], [27]. Within this region, ten populations of *P. officinalis* were randomly selected (Fig. 1). The distances separating the studied populations varied between 0.74 and 16.73 km (mean: 7.30 km). All populations were located in small forest fragments embedded within a hostile agricultural and urban landscape matrix. At the beginning of April 2012, the number of flowering individuals was counted for each population. Simultaneously, in each population ten individuals were randomly selected for nectar sampling, and from each individual five flowers were harvested, yielding a total of 50 flowers per population.

Within 24 h after harvesting, nectar was extracted using sterile 5-µl microcapillaries. Nectar of flowers from the same plant was pooled and diluted in 150 µl of sterile distilled H_2_O [18], yielding a total of 100 nectar samples. Since floral nectar usually contains high concentrations of sucrose and other sugars and can also contain high levels of inorganic ions, nectar dilutions (even in distilled H_2_O) are not hypotonic and both bacteria and yeasts have been shown to remain viable in nectar dilutions in distilled H_2_O for several months [18]. Subsequently, fifty microliters was plated on both trypticase soy agar (TSA; Oxoid) and yeast extract peptone dextrose agar (YPDA; Difco), representing a general growth medium for bacteria and yeasts, respectively. These media have been used successfully for isolating microorganisms from nectar previously [10]–[12], [18]. Plates were incubated at 25°C for 10 days. For each plate one colony was picked for each morphologically distinct colony type, and further subcultivated to obtain pure cultures [18]. In addition, a preliminary screen of several morphologically identical colonies from the same plate had revealed that they all belonged to the same species, illustrating the suitability of the used approach. The obtained bacterial and yeast isolates were stored at −80°C in trypticase soy broth (Oxoid) and yeast extract peptone dextrose broth (Difco) containing 37.5% glycerol, respectively.

### DNA Extraction, PCR Amplification and Sequencing

For each isolate, genomic DNA was extracted from five-day old cultures grown on either TSA (bacteria) or YPDA (yeasts) using the phenol–chloroform extraction method [28]. Samples were amplified in a reaction volume of 20 µl, containing 312.5 µM of each dNTP, 1.0 µM of each primer, 1.25 units TaKaRa ExTaq polymerase, 1× Ex Taq Buffer (Clontech Laboratories, Palo Alto, CA, USA), and 5 ng genomic DNA (as measured by a Nanodrop spectrophotometer). DNA amplification of the D1/D2 domain of the large subunit rRNA and 16S rRNA gene was performed using the primer sets NL1–NL4 [29]) and 27F-1492R [30] for yeasts and bacteria, respectively. When amplification failed using the latter pair, primers 1387R [31] or 1541R [32] were used as reverse primer. Before amplification, DNA samples were denatured at 94°C for 2 min. Next, 30 cycles were run consisting of 45 s at 94°C, 45 s at 55°C (for NL1–NL4) or 59°C (for 27F-1492R/1387R/1541R), and 45 s at 72°C, with a final extension at 72°C for 10 min. Sequencing was performed using the reverse primer used for DNA amplification.

### Data Analysis

The obtained sequences were compared with reference sequences using BLAST software [33] and the Ribosomal Database Project (RDP) website [34] (http://rdp.cme.msu.edu/). Isolates were assigned to the highest taxonomic rank possible (generally the species level) using the RDP classifier, BLAST analysis (uncultured/environmental sample sequences excluded), and based on the nearest neighbors in a phylogenetic tree containing GenBank sequences from several type strains showing the highest sequence homology to our sequences. To this end, both our sequences and the reference sequences were aligned with Clustal W implemented in MEGA5 [35], followed by trimming to consensus start and end motifs. Phylogenetic trees were calculated by the neighbour-joining method [36] implemented in Clustal X and displayed by TreeView version 1.6.6 [37]. Support of internal nodes was assessed using bootstrap analysis performed with 1000 replications. For ease of visualization, highly similar sequences (>99% sequence identity) were restricted to one representative sequence per OTU. In all cases, presumptive identifications based on top BLAST hits were confirmed by the nearest neighbor in the phylogenetic tree containing type strain sequences.

For subsequent analyses, bacterial and yeast OTUs were assigned using the Mothur v.1.23.1 software program [38]. DNA dissimilarity cut-offs of 1% and 3% were used in these analyses. For each OTU, the capability to grow in nectar was verified for a few isolates according [7], [18], [39]. All isolates tested were found to tolerate sucrose concentrations of at least 50% (w/v). In addition, all examined bacterial isolates showed catalase activity, suggesting that the detected OTUs are physiologically capable to overcome the presence of toxic hydrogen peroxide in nectar. Representative sequences for each OTU were deposited in GenBank (accession numbers KC433478–KC433527).

In order to assess the overall richness of microbial OTUs in the whole study region, sample-based rarefaction methods were applied to species presence-absence [40], [41]. Since the nectar of multiple flowers from a single plant was combined, individual plants rather than nectar drops were considered as sample units [12]. In this analysis, OTU occurrence data from all individuals were analyzed together, irrespective of the population of origin, yielding a rarefaction curve that assesses overall species richness of nectar yeasts and bacteria at the landscape scale. Rarefaction curves were computed using EstimateS version 8.2 [40], with 50 randomizations and sampling without replacement. Analyses were performed for bacteria and yeasts separately. Additionally, as our taxa richness data are based on incidence, the expected yeast and bacterial OTU richness in nectar was also determined using the nonparametric estimator Chao2 [42]. Rarefaction generates the expected number of species (OTUs) in a small collection of *n* samples drawn at random from the large pool of *N* samples [43]. In contrast, richness estimators predict the total richness of a community from samples [40].

For each population, OTU richness of bacteria and yeasts was determined by counting the total number of different bacterial and yeast OTUs. The observed richness was related to the size of the population using the Pearson product-moment correlation coefficient. Population size was log transformed prior to analysis. To visualize differences in microbial community structure among populations, we applied non-metric multidimensional scaling (NMDS) ordination techniques using the program PC-ORD version 6 [44]. As distance measure, we used the Bray-Curtis coefficient. This coefficient is also known as the Sørensen or Czekanowski coefficient and is considered as one of the most robust measures for this purpose [45]. In addition, pairwise similarity matrices were created to determine microbial community similarity between populations. The Jaccard index was used to describe the similarity in composition of the bacterial and yeast nectar communities [46]. For each population, the nearest distance (bird’s eye view) to any other population was also determined. A Mantel test was used to test the hypothesis that community similarity was related to the distance separating populations. Statistical significance was determined using 9999 randomizations in PopTools [47].

## Results

**Figure 2:**
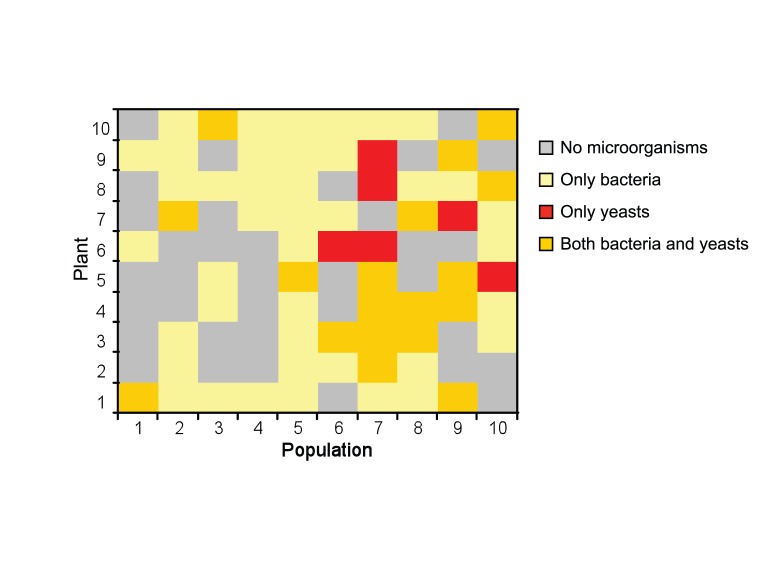
Distribution of culturable nectar yeasts and bacteria in floral nectar of ten plant individuals from ten *Pulmonaria officinalis* populations.

Bacterial and yeast isolates were obtained from both TSA and YPDA. Following isolation and purification, a total of 37 yeast and 152 bacterial isolates was obtained from nectar samples from 24 and 59 *P. officinalis* plants respectively, with 18 plants containing both yeasts and bacteria in their nectar (Fig. 2). For 35 out of the 100 nectar samples, no microbial growth was observed. Yeasts were recovered from nine out of ten populations, whereas bacteria were found in all sampled populations (Fig. 2).

**Figure 3:**
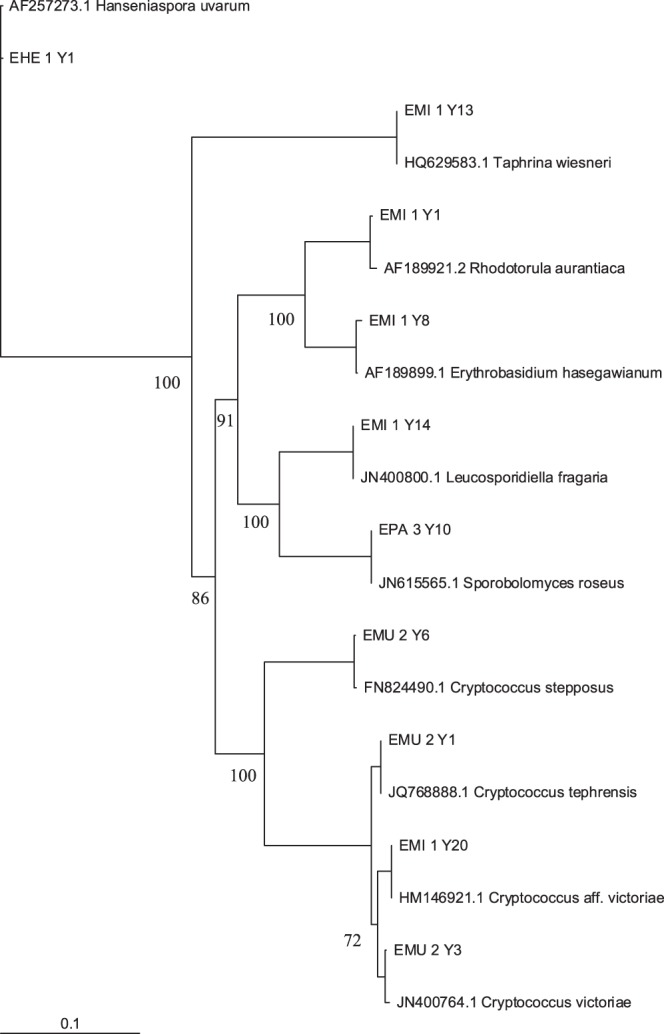
Neighbour-joining phylogram showing phylogenetic relationships between different large subunit rRNA gene sequences from *Pulmonaria officinalis* nectar-inhabiting yeasts and reference sequences of the most related type strains found in GenBank. Bootstrap percentages based on 1000 replications are shown at the major nodes.

**Figure 4:**
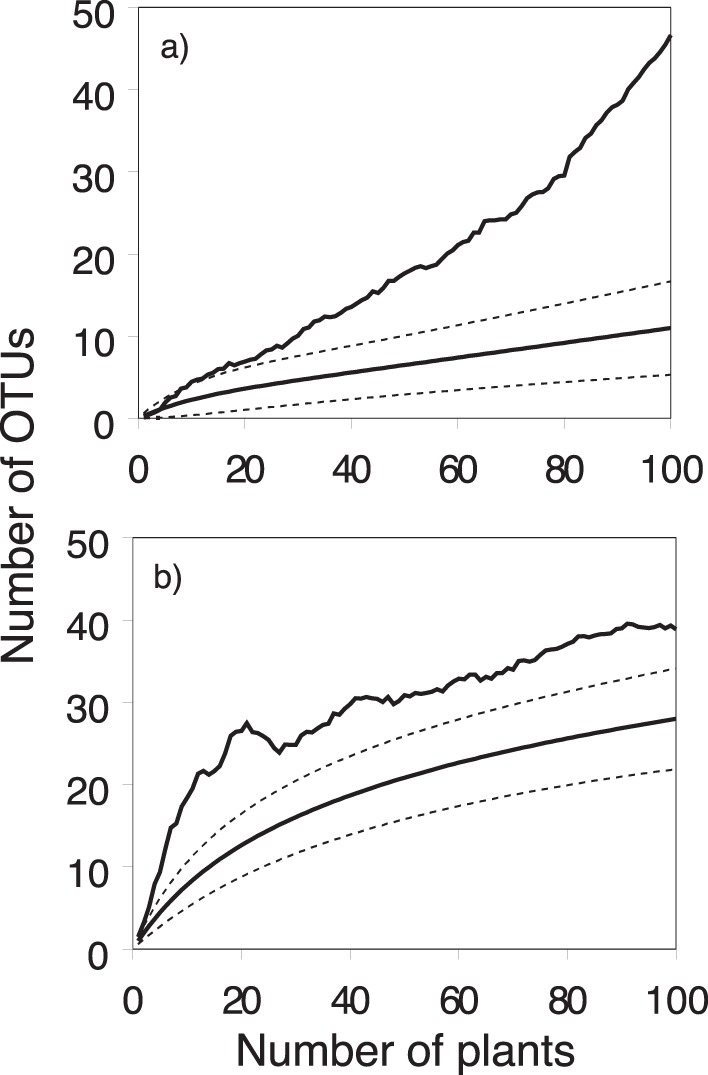
Rarefaction curves (bold, solid line) and the nonparametric estimator Chao2 (thin solid line) of microbial OTU richness found in the floral nectar of 100 sampled individuals of *Pulmonaria officinalis*. Dotted lines represent 95% confidence intervals. Rarefaction curves are given for a) yeast and b) bacterial OTUs (based on a DNA dissimilarity cut-off value of 1%).

Using a 3% sequence dissimilarity cut-off value, nine yeast OTUs (OTUs_0.03_) were identified, comprising both ascomycetous and basidiomycetous yeasts (Table 1). Two additional OTUs were identified when the dissimilarity cut-off was lowered to 1% (OTUs_0.01_), corresponding to 11 different yeast species (Table 1). These included for example *Metschnikowia reukaufii*, *Candida bombi*, *Sporobolomyces roseus* and several *Cryptococcus* species (Fig. 3). Rarefaction curves showed that the number of OTUs was relatively close to saturation (Fig. 4a). However, the nonparametric richness estimator Chao2 gradually shifted from the observed species richness, indicating that our sampling only detected a part of the total estimated yeast species richness. Most likely, the erratic behavior of the Chao2 estimator was caused by the overall low yeast abundance. *M. reukaufii* and *C. bombi* were recorded as the most common yeast species, occurring in five and three populations, respectively. All other yeast OTUs were only observed in a single population.

**Table 1:** Yeast operational taxonomic units (OTUs) identified in this study.

OTU_0.03_ a	OTU_0.01_ b	Representative isolate (GenBank Accession N°)	Phylogenetic affiliation^c^						Number of isolates^d^	Number of populations^e^
			Phylum	Family	Closest match in GenBank to identified species (Accession N°)	Sequence identity (%)	E-value	Score		
OTU_0.03_ Y1	OTU_0.01_Y1	5.5.2-Y1 (KC433521)	*Ascomycota*	*Myxotrichaceae*	*Geomyces pannorum* (JQ768405)	500/504 (99.2)	0.0	988.0	1	1
OTU_0.03_ Y2	OTU_0.01_ Y2	6.3.1-Y1 (KC433522)	*Ascomycota*	*Metschnikowiaceae*	*Metschnikowia reukaufii* (HM627129)	449/449 (100.0)	0.0	898.0	15	5
OTU_0.03_ Y3	OTU_0.01_ Y3	6.3.2-Y3 (KC433523)	*Ascomycota*	*Saccharomycetaceae*	*Candida cf. lactis-condensi* (AY257059)	430/431 (99.8)	0.0	852.0	1	1
OTU_0.03_ Y4	OTU_0.01_ Y4	7.9.2-Y1 (KC433526)	*Ascomycota*	*Saccharomycetaceae*	*Candida bombi* (AF406929)	429/430 (99.8)	0.0	850.0	10	3
OTU_0.03_ Y5	OTU_0.01_ Y5	1.1.1-Y1 (KC433517)	*Basidiomycota*	*Tremellaceae*	*Cryptococcus macerans* (FR716588)	500/500 (100.0)	0.0	1000.0	2	1
OTU_0.03_ Y6	OTU_0.01_ Y6	1.1.2-Y3 (KC433518)	*Basidiomycota*	*Tremellaceae*	*Cryptococcus tephrensis* (JQ768888)	502/502 (100.0)	0.0	1004.0	1	1
	OTU_0.01_ Y7	2.7.1-Y1 (KC433520)	*Basidiomycota*	*Tremellaceae*	*Cryptococcus* aff. *victoriae* (HM146921)	502/502 (100.0)	0.0	1004.0	1	1
	OTU_0.01_ Y8	6.6.1-Y1 (KC433524)	*Basidiomycota*	*Tremellaceae*	*Cryptococcus victoriae* (JN615569)	502/502 (100.0)	0.0	1004.0	2	1
OTU_0.03_ Y7	OTU_0.01_ Y9	1.1.1-Y3 (KC433519)	*Basidiomycota*	*Tremellaceae*	*Cryptococcus oeirensis* (FN357225)	505/505 (100.0)	0.0	1010.0	1	1
OTU_0.03_ Y8	OTU_0.01_ Y10	1.1.2-Y2 (KC433527)	*Basidiomycota*	*Tremellaceae*	*Holtermanniella takashimae* f (FR819696)	504/504 (100.0)	0.0	1008.0	2	1
OTU_0.03_ Y9	OTU_0.01_ Y11	10.5.1-Y1 (KC433525)	*Basidiomycota*	*Sporobolomycetaceae*	*Sporobolomyces roseus* (JN615565)	479/479 (100.0)	0.0	958.0	1	1

a Yeasts were grouped into OTUs defined by 97% sequence identity at the large subunit rRNA gene (430–505 bp).

b Yeasts were grouped into OTUs defined by 99% sequence identity at the large subunit rRNA gene (430–505 bp).

c Based on BLAST analysis (August 2012). Only closest matches to named species are reported.

d Number of isolates recovered in this study.

e Number of plant populations in which the corresponding OTUs were recorded.

f Recently described species, related to *Cryptococcus* species [49].

**Figure 5:**
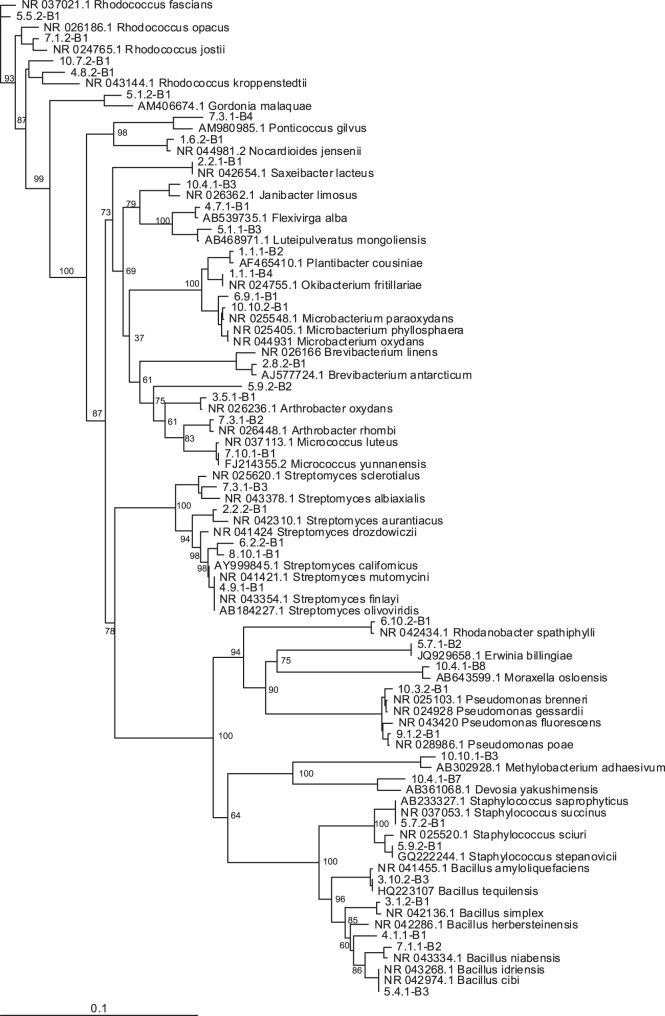
Neighbour-joining phylogram showing phylogenetic relationships between 16S rRNA gene sequences from *Pulmonaria officinalis* nectar-inhabiting bacteria and reference sequences of the most related type strains found in GenBank. For ease of visualization, the dataset was limited to one representative sequence (see Table 1 and 2) for each operational taxonomic unit (OTU) found in this study at a DNA dissimilarity cut-off value of 1%. Bootstrap percentages based on 1000 replications are shown at the major nodes.

Using a 3% cut-off value, a total of 28 bacterial OTUs (OTUs_0.03_) was detected (Table 2). By lowering the dissimilarity cut-off to 1% 11 additional OTUs were found, resulting in a total of 39 OTUs_0.01_ (Table 2). As for the yeasts, the rarefaction curves were relatively close to reach a plateau. However, in contrast with the yeasts, the Chao 2 estimator gave a predicted OTU richness which was close to the number of observed OTUs, resulting in an estimated richness of the nectar bacterial community of 39 OTU_0.03_ and 54 OTU_0.01_ (Fig. 4b). The recovered bacteria belonged to three major phyla, including *Actinobacteria* (18 OTUs_0.03_; 25 OTUs_0.01_), *Firmicutes* (4 OTUs_0.03_; 7 OTUs_0.01_) and *Proteobacteria* (*Alpha* and *Gamma* subdivisions; 6 OTUs_0.03_; 7 OTUs_0.01_) (Table 2). The most common bacteria were species from the genera *Rhodococcus*, *Microbacterium* and *Methylobacterium* which were retrieved in five or more populations (Table 2). Other OTUs that were identified (>97.5% sequence homology with GenBank sequence) included members from the genera *Arthrobacter*, *Bacillus*, *Brachybacterium*, *Brevibacterium*, *Devosia*, *Erwinia*, *Enhydrobacter*, *Flexivirga*, *Gordonia*, *Janibacter*, *Luteipulveratus*, *Micrococcus*, *Moraxella*, *Nocardioides, Okibacterium*, *Plantibacter*, *Ponticoccus*, *Pseudomonas*, *Rhodanobacter*, *Saxeibacter*, *Staphylococcus* and *Streptomyces* (Table 2; Fig. 5). Although the presence of *Micrococcus* and *Staphylococcus* may suggest possible contamination as these bacteria may also occur on the skin of humans and animals, we clearly showed that the detected species were able to resist high sugar concentrations typically experienced in nectar. In addition, members of *Staphylococcus* and the *Micrococcaceae* family have been isolated from other nectar sources as well [18], [19]. Therefore, we can reasonably assume that all bacteria obtained in our study can be considered as true nectar-inhabiting microbes.

**Table 2:** Bacterial operational taxonomic units (OTUs) identified in this study.

OTU_0.03_ a	OTU_0.01_ b	Representative isolate (GenBank Accession N°)	Phylogenetic affiliation^c^						Number of isolates^d^	Number of populations^e^
			Phylum	Family	Closest match in GenBank to identified species (Accession N°)	Sequence identity (%)	E-value	Score		
OTU_0.03_ B1	OTU_0.01_ B1	2.8.2-B1 (KC433478)	*Actinobacteria*	*Brevibacteriaceae*	*Brevibacterium antarcticum* (AJ577724)	737/740 (99.6)	0.0	1464.0	4	2
OTU_0.03_ B2	OTU_0.01_ B2	5.1.2-B1 (KC433502)	*Actinobacteria*	*Gordoniaceae*	*Gordonia malaquae* (AB546299)	730/737 (99.1)	0.0	1438.0	2	1
OTU_0.03_ B3	OTU_0.01_ B3	5.9.2-B2 (KC433511)	*Actinobacteria*	*Dermabacteraceae*	*Brachybacterium tyrofermentans* (NR_026272)	732/736 (99.5)	0.0	1452.0	5	3
OTU_0.03_ B4	OTU_0.01_ B4	4.7.1-B1 (KC433494)	*Actinobacteria*	*Dermacoccaceae*	*Flexivirga alba* (AB539735)	734/736 (99.7)	0.0	1462.0	2	2
OTU_0.03_ B5	OTU_0.01_ B5	5.1.1-B3 (KC433508)	*Actinobacteria*	*Dermacoccaceae*	*Luteipulveratus mongoliensis* (AB468971)	730/736 (99.2)	0.0	1442.0	4	2
OTU_0.03_ B6	OTU_0.01_ B6	10.4.1-B3 (KC433492)	*Actinobacteria*	*Intrasporangiaceae*	*Janibacter limosus* (DQ406852)	727/737 (98.6)	0.0	1416.0	2	1
OTU_0.03_ B7	OTU_0.01_ B7	1.1.1-B2 (KC433497)	*Actinobacteria*	*Microbacteriaceae*	*Plantibacter cousiniae* (JX133202)	737/738 (99.9)	0.0	1470.0	2	1
	OTU_0.01_ B8	1.1.1-B4 (KC433506)	*Actinobacteria*	*Microbacteriaceae*	*Okibacterium fritillariae* (NR_024755)	738/738 (100.0)	0.0	1476.0	1	1
	OTU_0.01_ B9	10.10.2-B1 (KC433493)	*Actinobacteria*	*Microbacteriaceae*	*Microbacterium paraoxydans* (JX293289.1)	735/738 (99.6)	0.0	1452.0	11	6
	OTU_0.01_ B10	6.9.1-B1 (KC433514)	*Actinobacteria*	*Microbacteriaceae*	*Microbacterium phyllosphaerae* (EF143429)	738/739 (99.9)	0.0	1468.0	5	4
OTU_0.03_ B8	OTU_0.01_ B11	3.5.1-B1 (KC433483)	*Actinobacteria*	*Micrococcaceae*	*Arthrobacter oxydans* (HQ236023)	737/738 (99.9)	0.0	1470.0	1	1
OTU_0.03_ B9	OTU_0.01_ B12	7.10.1-B1 (KC433482)	*Actinobacteria*	*Micrococcaceae*	*Micrococcus yunnanensis* (JQ659453); *M. luteus* (JX262404)	739/739 (100.0)	0.0	1478.0	2	2
OTU_0.03_ B10	OTU_0.01_ B13	7.3.1-B2 (KC433488)	*Actinobacteria*	*Micrococcaceae*	*Arthrobacter rhombi* (JF700476)	738/739 (99.9)	0.0	1472.0	4	4
OTU_0.03_ B11	OTU_0.01_ B14	2.2.1-B1 (KC433499)	*Actinobacteria*	*Nakamurellaceae*	*Saxeibacter lacteus* (HE599561)	736/736 (100.0)	0.0	1472.0	1	1
OTU_0.03_ B12	OTU_0.01_ B15	5.5.2-B1 (KC433510)	*Actinobacteria*	*Nocardiaceae*	*Rhodococcus fascians* (HE800820)	727/736 (98.9)	0.0	1426.0	16	3
	OTU_0.01_ B16	7.1.2-B1 (KC433513)	*Actinobacteria*	*Nocardiaceae*	*Rhodococcus jostii* (JQ828847)	730/736 (99.2)	0.0	1442.0	7	3
OTU_0.03_ B13	OTU_0.01_ B17	4.8.2-B1 (KC433501)	*Actinobacteria*	*Nocardiaceae*	*Rhodococcus kroppenstedtii* (JN873342)	721/732 (98.5)	0.0	1408.0	1	1
OTU_0.03_ B14	OTU_0.01_ B18	10.7.2-B1 (KC433516)	*Actinobacteria*	*Nocardiaceae*	*Rhodococcus opacus* (AP011115)	721/738 (97.7)	0.0	1386.0	11	5
OTU_0.03_ B15	OTU_0.01_ B19	1.6.2-B1 (KC433498)	*Actinobacteria*	*Nocardioidaceae*	*Nocardioides jensenii* (NR_044981)	731/735 (99.5)	0.0	1450.0	3	2
OTU_0.03_ B16	OTU_0.01_ B20	7.3.1-B4 (KC433487)	*Actinobacteria*	*Propionibacteriaceae*	*Ponticoccus gilvus* (JN712171)	718/736 (97.6)	0.0	1382.0	1	1
OTU_0.03_ B17	OTU_0.01_ B21	7.3.1-B3 (KC433489)	*Actinobacteria*	*Streptomycetaceae*	*Streptomyces albiaxialis* (FJ200397)*; S. sclerotialus* (AB184071)	736/744 (98.9)	0.0	1448.0	1	1
OTU_0.03_ B18	OTU_0.01_ B22	2.2.2-B1 (KC433500)	*Actinobacteria*	*Streptomycetaceae*	*Streptomyces aurantiacus* (FJ486316)	743/744 (99.9)	0.0	1482.0	1	1
	OTU_0.01_ B23	4.9.1-B1 (KC433507)	*Actinobacteria*	*Streptomycetaceae*	*Streptomyces* sp., incl. *S. finlayi, S. mutomycini, S. olivoviridis,…*	744/744 (100.0)	0.0	1375.0	2	2
	OTU_0.01_ B24	8.10.1-B1 (KC433490)	*Actinobacteria*	*Streptomycetaceae*	*Streptomyces californicus* (DQ462663)	737/744 (99.1)	0.0	1436.0	1	1
	OTU_0.01_ B25	6.2.2-B1 (KC433485)	*Actinobacteria*	*Streptomycetaceae*	*Streptomyces californicus* (DQ462663)	734/744 (98.7)	0.0	1426.0	1	1
OTU_0.03_ B19	OTU_0.01_ B26	3.10.2-B3 (KC433480)	*Firmicutes*	*Bacillaceae*	*Bacillus* sp., incl *B. subtilis, B. amyloliquefaciens,…*	736/736 (100.0)	0.0	1472.0	2	2
OTU_0.03_ B20	OTU_0.01_ B27	3.1.2-B1 (KC433479)	*Firmicutes*	*Bacillaceae*	*Bacillus simplex* (JX144722)	738/738 (100.0)	0.0	1476.0	1	1
OTU_0.03_ B21	OTU_0.01_ B28	4.1.1-B1 (KC433484)	*Firmicutes*	*Bacillaceae*	*Bacillus herbersteinensis* (EU867376)	734/738 (99.5)	0.0	1456.0	1	1
	OTU_0.01_ B29	5.4.1-B3 (KC433509)	*Firmicutes*	*Bacillaceae*	*Bacillus idriensis* (JQ956518); *B. cibi* (JN645994)	738/738 (100.0)	0.0	1476.0	2	1
	OTU_0.01_ B30	7.1.1-B2 (KC433512)	*Firmicutes*	*Bacillaceae*	*Bacillus niabensis* (JN208097); *B. licheniformis* (EU221362)	737/737 (100.0)	0.0	1474.0	1	1
OTU_0.03_ B22	OTU_0.01_ B31	5.9.2-B1 (KC433503)	*Firmicutes*	*Staphylococcaceae*	*Staphylococcus sciuri* (AM062696.1)*; S. stepanovicii* (GQ222243)	738/738 (100.0)	0.0	1476.0	3	1
	OTU_0.01_ B32	5.7.2-B1 (KC433481)	*Firmicutes*	*Staphylococcaceae*	*Staphylococcus* sp., incl. *S*. *saprophyticus, S. succinus, …*	738/738 (100.0)	0.0	1476.0	2	1
OTU_0.03_ B23	OTU_0.01_ B33	10.4.1-B7 (KC433504)	*Proteobacteria*	*Hyphomicrobiaceae*	*Devosia yakushimensis* (AB682141)	715/733 (97.5)	0.0	1376.0	1	1
OTU_0.03_ B24	OTU_0.01_ B34	10.10.1-B3 (KC433495)	*Proteobacteria*	*Methylobacteriaceae*	*Methylobacterium adhaesivum* (AB698722)	734/734 (100.0)	0.0	1468.0	10	7
OTU_0.03_ B25	OTU_0.01_ B35	5.7.1-B2 (KC433505)	*Proteobacteria*	*Enterobacteriaceae*	*Erwinia billingiae* (JQ929658)	737/737 (100.0)	0.0	1474.0	2	1
OTU_0.03_ B26	OTU_0.01_ B36	10.4.1-B8 (KC433515)	*Proteobacteria*	*Moraxellaceae*	*Moraxella osloensis* (JX293292)	738/738 (100.0)	0.0	1476.0	1	1
OTU_0.03_ B27	OTU_0.01_ B37	9.1.2-B1 (KC433496)	*Proteobacteria*	*Pseudomonadaceae*	*Pseudomonas poae* (HQ898911)	737/737 (100.0)	0.0	1474.0	7	4
	OTU_0.01_ B38	10.3.2-B1 (KC433491)	*Proteobacteria*	*Pseudomonadaceae*	*Pseudomonas brenneri* (JX417436)	737/737 (100.0)	0.0	1474.0	3	2
OTU_0.03_ B28	OTU _0.01_B39	6.10.2-B1 (KC433486)	*Proteobacteria*	*Xanthomonadaceae*	*Rhodanobacter spathiphylli* (NR_042434)	736/738 (99.7)	0.0	1466.0	1	1

a Bacteria were grouped into OTUs defined by 97% sequence identity at the 16S rRNA gene (732–744 bp).

b Bacteria were grouped into OTUs defined by 99% sequence identity at the 16S rRNA gene (732–744 bp).

c Based on BLAST analysis (August 2012). Only closest matches to named species are reported.

d Number of isolates recovered in this study. 24 isolates could only be identified to the family level, including members of the *Microbacteriaceae* (18), *Staphylococcaceae* (3), *Streptomycetaceae* (2) and *Pseudomonadaceae* (1).

e Number of plant populations in which the corresponding OTUs were recorded.

**Figure 6:**
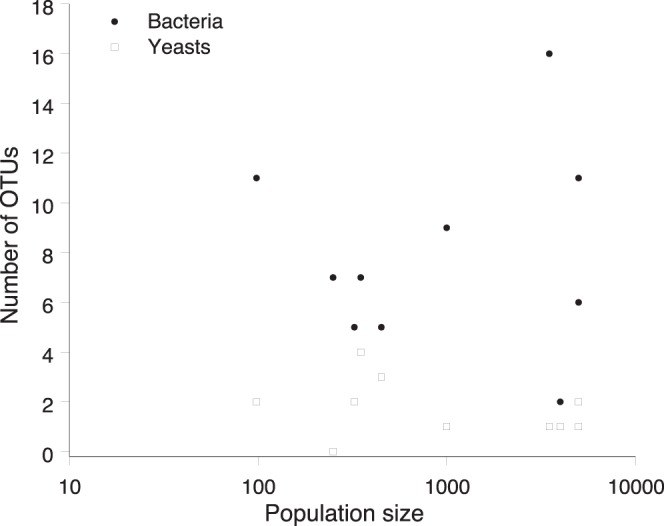
Relationship between population size (number of flowering ramets) and the number of bacterial and yeast OTUs (based on a DNA dissimilarity cut-off value of 1%) in the floral nectar of *Pulmonaria officinalis*.

**Figure 7:**
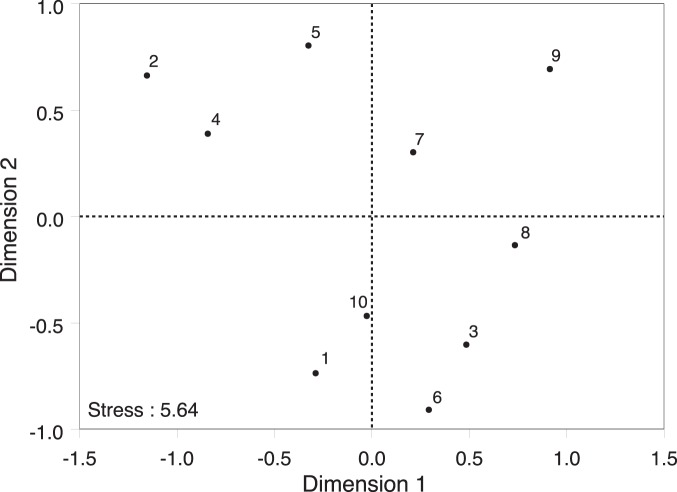
NMDS ordination of the total microbial community composition (bacteria and yeasts) in the floral nectar of *Pulmonaria officinalis* obtained from sampling flowers from ten individuals in ten populations. Numbers refer to the populations depicted in Fig. 1.

The sampled *P. officinalis* populations differed in size between 98 and >5000 flowering individuals. The number of yeast OTUs observed per population varied between 0 and 4 (mean: 1.7) (irrespective of the cut-off value used), and was not significantly related to population size (*r* = −0.18, *P*>0.05) (Fig. 5). The number of bacterial OTUs observed per population varied between 2 and 13 OTUs_0.03_ (mean: 6.9) and 2 and 16 OTUs_0.01_ (mean: 7.9), and was also not significantly related to the size of the plant population (*r* = −0.03, *P*>0.05) (Fig. 6). Although the NMDS analysis showed that some geographic clustering in microbial community (taking into account both bacteria and yeasts) was present (Fig. 7), the overall similarity in community composition was low (average Jaccard index: 0.14). Populations 7, 8 and 9 and populations 2, 4 and 5 formed distinct clusters on the NMDS graph which to some extent coincided with their geographic location in the landscape (Fig. 1). However, there was no significant relationship between community similarity and geographic distance (*r*_M_ = −0.22, *P* = 0.09).

## Discussion

### Microorganisms

In this study a wide variety of nectar-inhabiting microorganisms was found in the floral nectar of the early-flowering forest herb *P. officinalis*. Using a 1% dissimilarity cut-off, a total of eleven different yeast species was identified, many of which have been recorded in nectar before, including for example *M. reukaufii*, *C. bombi*, *C. victoriae*, *C. macerans*, and *S. roseus* [8], [12]. Although the number of species retrieved seems not to be unusual compared to results reported in similar studies [8], [12], it is surprising to see that within a single plant species almost as much yeast species were found as were reported in previously published datasets, which mostly covered a large number of plant species. For example, studying 128 nectar drops from 24 plant species in Spain yielded 216 yeast isolates and a total of 12 different yeast species [12]. Similarly, 11 different yeast species in 42 isolates were found in a total of 11 tropical plant species from the Yucatan Peninsula, Mexico [8].

There is less information about the occurrence and distribution of bacterial species within the floral nectar of individual plants and species. Recently, 53 bacterial isolates were recovered from 38 nectar samples (53.5% of all investigated nectar drops) from 27 plant species belonging to 13 plant families occurring in South Africa [18]. This yielded a total of 18 and 24 bacterial OTUs at the 3% and 1% 16S rRNA gene dissimilarity cut-off, respectively. In *P. officinalis*, in 59% of the sampled plants bacterial OTUs were found. However, the number of bacterial OTUs was about twice as large as that found in [18], i.e. 28 and 39 at 3% and 1% dissimilarity cut-off, respectively. Similar to the results of [18], all OTUs recovered belonged to only three bacterial phyla (*Actinobacteria*, and to a lesser extent to *Firmicutes* and *Proteobacteria*), confirming previous findings that microbial communities in nectar are characterized by low phylogenetic diversity. Using both catalase activity and sucrose tolerance tests we also showed that all recovered species can hydrolyze hydrogen peroxide and display high osmotolerance, and thus are able to overcome some of the main stressors found in floral nectar. Interestingly, whereas *Proteobacteria* were the dominant bacteria in South-African plants, about 50% of the retrieved OTUs belonged to *Actinobacteria* in *P. officinalis*. The most prevalent genera of this phylum found in this study included *Microbacterium*, *Rhodococcus* and *Streptomyces*. Species belonging to these genera are known to thrive in a broad range of environments, including soils and many plant-associated environments such as roots and leaves. However, as far as we know, these bacteria have not been associated with nectar so far.

### Species Richness

In 35 individuals no culturable microorganisms were found, which may suggest that these plants were either not visited by insects [7], that no transfer of microorganisms that can survive and develop in nectar had occurred during flower visits [15], or that the present microbiota represented only non-culturable microorganisms. The latter, for example, could be assessed using culture-independent methods such as 454 pyrosequencing. These methods generally have a higher resolution compared to culture-based approaches, which are restricted to the isolation of culturable microorganisms. So far, however, no comparison has been made between conventional methods and culture-independent methods for studying nectar microbiota. A major drawback of culture-independent methods, on the other hand, is the fact that no isolates are available to investigate or confirm specific features that allow these microorganisms to survive and grow in nectar [7], [18], [38]. We further found that most plants contained only nectar bacteria (41%), while fewer plants were found containing both bacteria and yeast (18%) and only a minority containing only yeasts (6%). These results indicate that at least in this plant species bacteria may be much more widespread in nectar than yeast species and contrast with findings of Álvarez-Pérez and Herrera [48], who recently showed that bacteria and yeasts generally coexisted in floral nectar of a selection of wild Mediterranean plants.

The number of yeast species per plant population was low (on average 1.7 OTUs per population), indicating that only a few yeast species dominated in a population. Low diversity of yeast species is in line with previous studies investigating yeast diversity in individual floral nectar samples [8], [10], [12]. For example, in two populations of the winter-blooming herb *Helleborus foetidus* the nectar was dominated by a single yeast species (*M. reukauffi*), although several different yeast species were observed on the bodies of visiting insects [15]. The dominance of a particular yeast species in the floral nectar of plant species has been explained by filtering mechanisms, such as priority effects, which predict that early-arriving species have a competitive advantage toward late-arriving species. Using laboratory experiments, priority effects appeared to be important in structuring microbial communities in floral nectar [11]. However, results depended strongly on the phylogenetic relationships of the yeast strains involved. Priority effects were particularly strong between closely related species, whereas effects were less pronounced for phylogenetically distantly related species. Overall, these results suggest that yeast species can outcompete other species, and that the first species to colonize and spread within a plant population can become the dominant yeast in the population. Nevertheless, in contrast to Herrera et al. [15], who found yeasts in 72.5% of the investigated *H. foetidus* nectar samples, in our study yeasts were found in less than 25% of the *P. officinalis* individuals tested, suggesting that other factors such as chemical nectar composition may also play an important role in the distribution of nectar yeasts in *P. officinalis*. Clearly, more research is needed to investigate whether priority effects really are the dominant factor determining yeast community organization in this species, or whether the occurrence of yeast species is affected by the presence of bacteria, and vice versa [48].

### Community Turnover

We found low community similarity and no significant turnover in microbial community composition among populations. These results may either suggest that little exchange of microorganisms between populations occurred or that nectar conditions differed between populations thereby selecting for different microbial communities. These results are in line with genetic work that has shown a strong genetic differentiation between *P. officinalis* populations [23]. Alternatively, because the local community of co-flowering plant species also differed substantially between populations, this may additionally have affected the species composition of microorganisms potentially colonizing *P. officinalis* flowers. Since nectar is considered to be initially sterile [7], pollinators are believed to be the main vectors transferring micro-organisms from one plant to the other, and between populations [14], [15].

Given that the most common pollinators of *P. officinalis* in the study region are generalist pollinators [27] that visit several co-flowering species, it is reasonable to expect that microbial community composition in the floral nectar of *P. officinalis* populations reflects to some extent local plant species composition. Although it is likely that pollinators (most often bumblebees and bees) can cross smaller distances across agricultural landscapes, it is unlikely they fly across very large distances further contributing to the low similarity in species composition. Recent findings of Belisle et al. [14] have shown that non-random small-scale foraging of pollinators resulted in non-random distributions of nectar-inhabiting yeasts in the hummingbird-pollinated *Mimulus aurantiacus*, but it is unlikely that this foraging behavior contributes substantially to large-scale patterns of community turnover. The observed strong genetic differentiation between *P. officinalis* populations and significant isolation-by-distance [23] support this hypothesis.

### Conclusion

Nectar of the bee-pollinated forest herb *P. officinalis* was commonly colonized by microorganisms, both bacteria and to a lesser extent yeasts. However, large variation in community structure was observed between populations. The inability of pollinators to cross larger distances across hostile agricultural and urban landscapes has probably contributed to the observed low similarity in community composition. However, the importance of variation in nectar properties between populations or differences in local species composition of co-flowering plants as drivers of microbial community composition cannot be ruled out. Especially in plant species that are pollinated by generalist pollinators that visit several co-flowering species at the same time, local microbial community structure cannot be studied independently from the local plant community. These findings thus suggest that the assembly of the nectar microbiota is context-dependent. More research, both experimental and observational studies, is therefore needed to elucidate the ecological mechanisms explaining variation in microbial community structure within and among populations and to disentangle the importance of local and regional factors.
